# Screening and care for alcohol use disorder in France: expectations, barriers and levers using a mixed-methods approach

**DOI:** 10.1186/s12889-020-08495-x

**Published:** 2020-03-18

**Authors:** Marie Costa, Tangui Barré, Marion Coste, Issifou Yaya, Cyril Berenger, Marc Tanti, Christophe Cutarella, Marion Mora, Pierre Poloméni, Marianne Maynard, Danielle Teuma, Michaël Bazin, Gwenaelle Maradan, Perrine Roux, Patrizia Maria Carrieri

**Affiliations:** 1 Aix Marseille Univ, INSERM, IRD, SESSTIM, Sciences Economiques & Sociales de la Santé & Traitement de l’Information Médicale, Marseille, France; 2 ORS PACA, Observatoire régional de la santé Provence-Alpes-Côte d’Azur, Marseille, France; 3 Centre d’Epidémiologie et de Santé Publique des Armées (CESPA), Marseille, France; 4 Clinique Saint-Barnabé, Marseille, France; 5 Assistance Publique, Hôpitaux de Paris, Paris, France; 6 CRC Groupement Nord, Hôpital de la Croix Rousse, 69317 Lyon cedex 04, France; 7 Unité d’addictologie, Hôpitaux du Bassin de Thau, 34200 Sète, France; 8 Unité d’addictologie, Centre Hospitalier d’Allauch, 13190 Allauch, France

**Keywords:** Alcohol use disorder, Access to care, Mixed-methods, Survey, Semi-structured interviews, Textual analysis, France

## Abstract

**Background:**

The widespread under-screening and under-treatment of alcohol use disorder (AUD) contributes to its health and socioeconomic burden. We conducted a mixed-methods (qualitative and qualitative) study in people with alcohol use disorder (PWAUD) to explore their expectations, as well as barriers and levers to AUD care.

**Methods:**

Individuals with AUDIT > 15 (*N* = 179) were interviewed using computer-assisted interviews in several medical and non-medical sites (e.g., bars) (quantitative substudy). We also conducted semi-structured face-to-face interviews with 36 PWAUD (qualitative substudy). Using logistic regression, we explored factors associated with having previously received/sought care for AUD. Three major themes were identified in the qualitative textual analysis using a descending hierarchical classification.

**Results:**

Not socializing with heavy drinkers (AOR [95%CI]:3.84[1.66–8.85]), regular smoking (9.72[3.91–24.15]) and feeling discriminated against (2.35[1.10–5.05]) were independent levers to having sought/received care for AUD, while being aged < 50 and employment were independent barriers. The five predominant themes in PWAUD discourses emerging from the textual analysis were: drinking context, medical care, alcohol treatment, tobacco/addiction and family. When triangulating results from the logistic regression and the textual analysis, two barriers (social drinking and difficulties with the medical care system), and two levers (family influence and tobacco addiction), emerged.

**Conclusion:**

These results underline the need for interventions targeting families and the social network to increase awareness about AUD and related care. Simplified and novel comprehensive care trajectories are urgently needed to reduce the clinical and public health burden of AUD.

## Background

Alcohol Use Disorder (AUD) is defined as “*a chronic relapsing brain disease characterized by compulsive alcohol use, loss of control over alcohol intake, and a negative emotional state when not using*” [1]. AUD concerns approximately 7% of adults in France, which represents approximately 3.5 million people [2]. Almost 10% of French adults drink daily, 5% report binge drinking at least once a week, and 3.8% (approximately 2 million people) report regular alcohol intoxication [3]. In 2015, AUD was responsible for 41,000 deaths in the country [4]. With 22% of fatal road accidents involving drivers with illegal blood-alcohol levels, alcohol consumption in France remains a serious public health concern which also affects non-drinkers [5, 6]. AUD is an important socio-economic burden on the country, despite a slight decreasing trend in consumption in recent decades. The social cost - defined as the total monetary and non-monetary cost because of alcohol use - was 120 billion euros in 2010 in France [7].

One study conducted in the French general population highlighted that fewer than half those identified with AUD had sought mental healthcare in the previous 12 months and that, overall, approximately 10% of people with alcohol use disorder (PWAUD) in the country receive related medical care [8]. Linking more PWAUD to healthcare constitutes a major clinical and public health challenge. The following individual and contextual factors have been identified as barriers to receiving care for PWAUD in Europe: i) many people are not aware that they have unhealthy alcohol use [9]; ii) screening for AUD in primary care is rare [10, 11]; iii) a great deal of stigma is attached to AUD (despite alcohol being strongly integrated into several European cultural patterns) [12]; iv) many PWAUD do not wish to be totally abstinent [13]. With regard to the latter point, it is important to underline that reduced drinking is only slowly becoming an acceptable therapeutic goal in AUD care in France for health professionals and PWAUD [14–16]^.^ Until recently the standard AUD care was based on completely stopping alcohol consumption, maintaining abstinence, preventing chronic complications related to excessive alcohol consumption, and managing withdrawal symptoms. The recent licensing of the two new pharmacotherapies nalmefene and baclofen in France, confirms this change [17].

In a large European cross-sectional study [13] which explored individual barriers to seeking care, in addition to the four barriers mentioned above, “wanting to cope alone” was cited by 20.9% of the 1008 participants. Another study showed that people who had already experienced adverse health and social consequences due to AUD were more likely to seek help [18]. Furthermore, female gender [19] and psychiatric comorbidities [20] were associated with seeking care for AUD in a previous French study [8]. However, none of the studies mentioned above used a mixed-methods approach to explore PWAUD expectations of AUD care, or the barriers and levers to AUD care**.**

Patterns of consumption, beliefs, and representations about alcohol make it difficult to implement effective prevention in France. For example, the tradition and history linked to the production and consumption of wine create a positive image of the product which leads to related harms being minimized [21]. In terms of consumption patterns, unlike some other European countries where alcohol is mainly consumed at the weekend, in France, consumption is more spread out over the entire week [22]. Alcohol consumption is also a cultural and social norm in France, ingrained as early as adolescence [23]. It is regularly consumed during daily social interaction [24].

In addition to this, the alcohol industry employs a large number of people in France. For this reason, the French government finds it difficult to totally discredit this drug [25]. Recently, the French president reaffirmed his love for wine and declared that he drinks it at every meal [26]. Initiatives like “dry January”, a public health campaign encouraging alcohol abstinence throughout the month of January [27], have not yet been implemented in France. Furthermore, currently there is no intention to increase alcohol taxes unlike, for example, in the UK.

Increased knowledge about individual and structural barriers and levers, as well as PWAUD expectations of AUD care, will not only improve prevention, screening and care, but will also reduce the clinical and public health burden of AUD. The present mixed-methods study aimed at identifying individual and contextual factors associated with having sought/received care among PWAUD in France. The study was urgently needed for two main reasons: 1) the 2014 French Health Barometer survey, which explores AUD and other various health issues, did not study the dynamic of PWAUD exclusion from care; 2) Data documenting AUD screening and management in France, especially since the licensing of the new pharmacotherapies mentioned above, is scarce.

## Methods

### Aim

This study aimed to explore barriers and levers to accessing AUD care for people in France, and to better understand the individual and contextual factors which explain why only a small percentage of PWAUD receive medical follow-up for this disorder. This use of a convergent parallel design [28] was chosen to obtain a more comprehensive view of the phenomenon of access to AUD care.

### Design

ASIA (Access to Care and Indifference toward Alcohol, *Accès aux Soins et Indifference à l’Alcool* in French) is a multi-site, cross-sectional convergent parallel mixed-methods study [29].

### Ethics approval process

The ASIA study protocol was submitted for approval to the French Health and Medical Research National Institute Ethics and Evaluation Committee. After obtaining approval from the latter, it was submitted to and approved by the French Advisory Committee on the Treatment of Information in Health Research, which guarantees participants’ anonymity and confidentiality.

### Setting of the study

The study was conducted in France between 2016 and 2018.

### Inclusion criteria

For both substudies (i.e., quantitative and qualitative), the following inclusion criteria were used: aged over 18, French speaking, and Alcohol Use Disorder Identification Test (AUDIT) score > 15 (see below for details about this measure).

### Quantitative survey

#### Recruitment and data collection

Recruitment for the quantitative study took place in several sites: 1 clinic, 2 hospital-based addiction services, 4 hospital-based addiction care services (ELSA), and 2 bars. None of the participants recruited in ELSA or bars were receiving AUD care at enrolment. We chose these specific recruitment sites for three reasons: the target population (i.e. treated and untreated PWAUD), the diversity in profiles of participants they offered, and geographic proximity.

Two trained interviewers conducted computer-assisted interviews using a survey of up to 48 questions, which included the French version of the AUDIT questionnaire at the beginning of the survey. Survey data capture was performed using sphinx software. In all recruitment sites, the interviewers approached potential participants and presented the study to them. In the 2 bars, interviewers approached everyone they met. All recruitment was carried out on a voluntary basis.

The survey itself was presented on a tablet not on paper. The AUDIT score was immediately calculated as participants responded. If an AUDIT score ≤ 15 was calculated, the survey ended automatically. Participants with an AUDIT score > 15 were able to complete the survey. Of the 225 people approached, 31 refused to participate and 14 did not meet inclusion criteria.

#### Consent to participate

People who agreed to participate in the quantitative study provided written consent and signed a study information form before they took the survey.

#### Study population

Among the 180 individuals subsequently interviewed, one did not respond to the question “Have you ever sought or received care to reduce or stop your alcohol consumption?” and was secondarily excluded. Therefore our study population comprised 179 individuals.

#### Outcome and potential correlates

The main outcome was built using the question “Have you ever sought or received AUD care in order to reduce or stop your alcohol consumption?”

Various potential variables were considered for the analysis (Table 1). These included socio-demographic characteristics, alcohol consumption, Etiam score, tobacco (dichotomised into ‘Regular/No’) and other drug use. Furthermore, the quality of participants’ relationships with their family physician was assessed using a question with 4 possible answers (“very good”, “good”, “poor” or “very poor”). Discrimination related to alcohol consumption was assessed using two questions: “Have you ever felt discriminated against because of your alcohol consumption?” (Yes/No), and for those answering Yes, “In which of the following contexts: hospital/general practitioner/family/at work/ everyday life?” Participants were also asked whether they were aware that AUD care no longer necessitates abstinence (Yes/No). The occurrence of alcohol-related health problems was assessed using the question “Have you experienced medical problems because of alcohol consumption?” (three possible answers: None / Slight problems which did not require medical intervention / Serious problems which required medical intervention).

**Table 1:** ASIA Questionnaire

**I. General information**		
**Question**	**Possible answers**	**Additional details**
1. Gender	Male/Female	
2. Education	High school diploma/Bachelor’s degree/Postgraduate degree	
3. Children	Yes/no	Do they live with you?
4. Do you live …?	Alone/ in a couple/ with other adults/ other	
5. Do you have a job?	Yes/no	What kind of contract do you have? Permanent contract, fixed-term contract/temporary worker/self-employed/other: No: Student/Retired/Jobseeker/other:
6. Employment	Unemployment benefit/ Disability allowance/scholarship/ retirement pension	
7. Do you live in …?	Own house/friend’s house/parents’ house/homeless shelter	
8. Housing conditions	Very uncomfortable / Uncomfortable / Comfortable / Very comfortable	
9. In the last 3 years, have you consulted a dentist?	Yes/no	
10. Do you wear glasses?	Yes/no	In the last 3 years, have you consulted an ophthalmologist? (Yes/no)
11. Do you have access to the internet?	Yes/no	
12. Do you have a means of transport (car, bike, public transport network nearby, etc.)?	Yes/no	
**II. Alcohol consumption**		
1. Do you think that your alcohol consumption could harm your health?	Yes/no	
2. Do you feel a physical / psychological craving when you do not consume alcohol?	Yes/no	
3. Have you ever had any health problems related to your drinking?	No/Yes, slight problems which decreasing my consumption resolved/ Yes, serious problems that required medical treatment (medicines, surgery, etc.)	
4. How long have you been at your current level of alcohol consumption?	Less than 1 year / 1 to 3 years / 3 to 5 years / 5 to 10 years / Over 10 years	
5. ETIAM SCALE [30] In the last week, how would you describe your desire to drink (0 corresponding to no desire to drink and 5 to a very strong desire to drink)	a) ... when you wake up?	0 1 2 3 4 5
	b) ... in the morning, between waking up and lunch?	0 1 2 3 4 5
	c) ... at lunch?	0 1 2 3 4 5
	d) ... in the afternoon before 5 pm?	0 1 2 3 4 5
	e) ... after 5 pm and before dinner?	0 1 2 3 4 5
	f) ... at dinner?	0 1 2 3 4 5
	g) ... in the late evening after dinner?	0 1 2 3 4 5
**III. Consumption of other drugs**		
1. In the past 3 months, have you used any of the following psychoactive substances? (Several choices possible)	Opiates (heroin) / Cocaine / Cannabis / Synthetic Drugs (MDMA, ecstasy, etc.)	For each substance cited, the frequency is requested Less than once a month / Once a month / Once a week / Almost every day
2. In the past 3 months, have you taken benzodiazepines (anxiolytics: Valium®, Xanax®, Lexomil®)	Yes/no	Outside of medical prescription / As part of a medical prescription, respecting the dosage and/or duration of treatment / As part of a medical prescription, exceeding the dosage and/or duration of treatment
3. Do you smoke tobacco?	Yes, regularly / Yes, a cigarette from time to time / No, but you smoked regularly in the past / No, never	
**IV. Experiences and feelings related to alcohol consumption**		
1. In general, what sensations does drinking give you?	Euphoria / Well-being / Appeasement / Decreased Anxiety / Decreased Sadness / Disinhibition / Self-Confidence / Feeling time goes faster / Sleep / Shame / Depression / Aggressiveness / Depression / Suicidal thoughts / Anxiety / Boredom / None / Other:	
2. Do you socialize with heavy drinkers?	Yes/no	Do they live with you?
3. In general, in which context (s) do you consume alcohol?	Alone / With your spouse, companion / At work / At home / With friends / In a bar / In public places (park, street, etc.) / In a restaurant / When going out to a party (nightclub, etc.) / Other:	
4. Have you ever felt discriminated against because of your drinking? (By discriminated we mean that you did not receive the same treatment / reception / attention as another person).	Yes/no	In the hospital / At the doctor’s / In my daily life (friends, shops, institutions, etc.) / In my professional environment / In my family / Other(s):
**V. Objectives/Aims concerning alcohol consumption**		
1. Would you like to change your alcohol consumption?	Yes/no	Reducing your consumption/Becoming sober/Controlling your consumption
2. Are you aware that you can receive medical care for alcohol misuse even if you do not wish to be completely sober?	Yes/no	
**VI. Care received**		
1. Do you have a doctor (usual doctor, family doctor)?	Yes/no	
2. How would you describe your relationship with your doctor?	Very good / Good / Not bad / Poor / Very poor	
3. Have you ever talked about your drinking with your doctor?	Yes, of his/her initiative / Yes, of my initiative / No	
4. Have you ever sought or received medical help to try to stop or reduce your alcohol consumption?	Yes, in the last 3 years / Yes, more than 3 years ago / Never	If never: You do not need it / You do not feel like it / You are scared / You do not have enough money / You live too far away from a hospital / doctor / health facility / You do not want to do not want to be sober / You do not feel able / You do not have enough time / You do not know who to talk to / You are ashamed to talk about it / You do not trust the healthcare system / You previously had a very negative experience / You did seek help, but did not receive the help you expectedp

#### Socio-demographic characteristics

Socio-demographic characteristics included gender, age, educational level (< vs. ≥ high-school diploma), having children, living in a couple (vs. alone), employment status and housing conditions.

#### Measuring alcohol consumption and craving intensity

Promoted by the World Health Organization, the Alcohol Use Identification Test (AUDIT) [31] is a 10-item questionnaire providing a score from 0 to 40. Scores between 8 (7 for women) and 15 suggest hazardous alcohol consumption, 16 to 19 harmful alcohol consumption, and > 20 alcohol dependence [32]. Accordingly, in the ASIA study, we chose to include only people with an AUDIT score > 15 because above this value, interventions are recommended [33]. We used the French version of AUDIT, which has been validated elsewhere [34].

Former PWAUD who successfully received care were not included. This was intentional so that our results would reflect the current evolving French context.

The Etiam (*Echelle Temporelle d’Intensité de l’Appétence Moyenne*, in English “the craving temporal intensity scale”) is a validated French scale which assesses the intensity of craving, on a score from 0 to 5, over seven 24-h periods (total score from 0 to 35). (Table 1, II.5) [30].

#### Tobacco status and other drug use

Tobacco status was assessed using the question “Do you smoke tobacco?” (Yes, regularly / Yes, a cigarette from time to time / No, but you smoked regularly in the past / No, never). The use of other drugs (Opiates/ Cocaine / Cannabis / Synthetic Drugs) over the previous 3 months was also assessed.

### Statistical analysis

A logistic regression model was used to identify factors associated with the outcome. Variables with a *p*-value < 0.2 in bivariate analyses were considered eligible for the multivariable analysis. When there were missing values for an explicative variable included in the multivariable analysis, the corresponding observations were excluded. A backward selection procedure was then implemented for eligible variables. Variables were considered significantly associated with the outcome if the *p*-value was ≤0.05. To check for collinearity, we computed the variance inflation factor (VIF) for the predictors. All analyses were performed using Stata 14.2 software.

### Qualitative study

#### Recruitment and procedures

For the qualitative study, between June 2016 and May 2017, a trained investigator conducted semi-structured in-depth interviews with 36 PWAUD to obtain a detailed description of their alcohol consumption history and associated experience with care (if applicable) from the PWAUD point of view. Recruitment took place in 4 medical structures in the south of France: a substance use disorder clinic, 2 CSAPA (Centre for Addiction Prevention, Support and Care), and an information and care centre for HIV (CISIH). We included these services in order to reach participants with different profiles. Some patients recruited through CSAPA were under legal obligation to have treatment because they had been arrested for driving under the influence of alcohol. Patients recruited in the CSAPA and CISIH services were not receiving AUD care at enrolment.

Medical staff informed their patients of the study and introduced potential participants to the study investigator. Five people refused to participate (2 mentioned a lack of time while 3 indicated not feeling comfortable with talking about the topic). Data saturation occurred after 36 interviews as no new themes emerged.

#### Consent to participate

People who agreed to participate in the qualitative study provided written consent and signed a study information form starting the interview.

#### Qualitative interviews

The study investigator performed face-to-face interviews in a private office to ensure confidentiality. The interview guide is presented in Table 2. Interviews were recorded and then transcribed using Microsoft Word, 2013.

**Table 2:** Semi-structured PWAUD interview guide

**Qualitative study among PWAUD**	
**Standard data collected**	Audit score Enrolment site Year of birth Gender Personal situation (e.g., living in a couple, children, etc.) Professional situation (e.g., employment status, retired, etc.) Substance use behaviour
**Semi-structured PWAUD interview guide**	
**Opening question**	You are experiencing difficulties with your alcohol consumption. I imagine it is difficult to deal with that. Could you tell me about your experience with alcohol since you started drinking, including, if applicable, your experience with health professionals for this specific problem?
**If the interviewed person does not mention these topics spontaneously, the interviewer must do so.**	➢ How long have you been drinking, how did it start? ➢ How did you realize that you had a drinking problem? ➢ Who have you talked to about your drinking problem? ➢ Have you talked about it with your family physician or another physician? ➢ What do you think prevents you from reducing or stopping your alcohol consumption? ➢ What are your goals: to stop drinking altogether? To drink less? ➢ What steps have you taken to try to change your consumption? (treatment, consultation with specialists, alternative medicines, etc.) ➢ Have you ever taken medication to stop drinking? Which medication(s) precisely? ➢ Have you looked for information concerning treatments to stop or reduce alcohol consumption? ➢ Have you heard of baclofen? Of nalmefene? ➢ Do you visit medical websites and / or forums? Does this help you? How?

#### Textual analysis

A descending hierarchical classification was performed on the whole corpus of interview transcriptions using the software package Iramuteq (*Interface de R pour les Analyses Multidimensionnelles de Textes et de Questionnaires*) (Version 0.7 Alpha 2). This software ensures accurate analyses for qualitative health research and is particularly suitable for voluminous corpora. More specifically, the descending hierarchical classification performs calculations on the co-occurrence of words in segments of text [35]. The results come in the form of speech classes that are described from the lexicon which is statistically overrepresented in the segments grouped in the classes. Each of the lexical classes can be described in detail by the lexicon that defines it, the characteristic segments that represent it, and associated variables [36].

#### Data triangulation

The triangulation of the results from the two substudies (i.e., quantitative and qualitative) was performed after data collection and analysis. MC, PMC, MC2 and TB took part in the data triangulation. Researchers first worked on data triangulation separately and then jointly discussed their individual analysis. MC wrote the first draft of the results section on triangulation. MC2, PMC and TB reviewed and corrected the section.

## Results

### Quantitative substudy results

#### General characteristics of study population

The socio-demographic characteristics of the quantitative substudy population are provided in Table 3. With respect to the AUDIT questionnaire, 82.1% of the sample drank alcohol more than four times a week, 9.5% two to three times a week, and 7.3% two to four times a month. On a typical day when alcohol was consumed, 31.3% of the sample consumed 10 standard drinks (each containing 10 g of alcohol), 25.1% seven or eight drinks, and 30.7% five or six drinks. The median AUDIT score was 29 (IQ: 23–33). One hundred and nine participants (60.9%) had already sought or received care to stop or reduce their alcohol consumption.

**Table 3:** Sociodemographic characteristics of the quantitative substudy population

	Having never sought or received care N (%)	Having already sought or received care N (%)	Total N	*P*-value (chi-squared test)
**Gender**				
Male	43 (37.07)	73 (62.93)	116	0.449
Female	27 (42.86)	36 (57.14)	63	
**Age**				
> = 50 years	14 (25.45)	41 (74.55)	55	0.013
< 50 years	56 (45.16)	68 (54.84)	124	
**Education**				
≥ High-school diploma	53 (46.09)	62 (53.91)	115	0.036
< High-school diploma	16 (26.23)	45 (73.77)	61	
Missing data	1 (33.3)	2 (66.6)	3	
**Having children**				
Yes	26 (28.89)	64 (71.11)	90	0.011
No	44 (50.0)	44 (50.0)	88	
Missing data	0 (0)	1 (100)	1	
**Living alone**				
Yes	25 (32.47)	52 (67.53)	77	0.114
No	45 (44.12)	57 (55.88)	102	
**Employment**				
Having a job	46 (49.46)	47 (50.54)	93	0.003
Not having a job	24 (27.91)	62 (72.09)	86	
**Housing conditions**				
Owner	54 (39.71)	82 (60.29)	136	0.617
Friend’s house	5 (41.67)	7 (58.33)	12	
Parents’ house	9 (42.86)	12 (57.14)	21	
Homeless shelter	1 (12.5)	7 (87.5)	8	
Missing data	1 (50)	1 (50)	2	

#### Types of AUD care sought /received

In terms of care for AUD, 7.3% of participants in the quantitative study sample were currently receiving pharmacotherapy, 10% were in hospital day care, 8.4% were consulting an addiction specialist, 12.3% were consulting a psychologist or psychiatrist, 1.1% were participating in support groups, 0.5% were participating in online discussions, 1.6% were attending an alcohol-related association, and 1.1% were frequenting Alcoholics Anonymous.

#### Bivariate analyses

The results of the bivariate analyses are shown in Table 4. People over 50 years old, those without at least a high-school certificate and those with children were all more likely (*p* < 0.05) to have already sought or received care to reduce or stop their alcohol consumption (i.e., the outcome). In the bivariate model, having been discriminated against because of alcohol consumption, not socializing with heavy drinkers, being a regular smoker and being a heavy binge drinker were all associated with a greater likelihood of seeking/receiving care. Conversely, having a job was associated with a lower likelihood of seeking/receiving care.

**Table 4:** Correlates of having sought or received care to reduce or stop alcohol consumption, quantitative study, *n* = 179

		Bivariate			Multivariable		
	N (%)	OR	95% CI	*p*-value	aOR	95% CI	*p*-value
**Gender**							
Female	63 (36)	**1**					
Male	116 (73)	**1.27**	0.68–2.38	0.449			
**Age**							
< 50	124 (68)	**1**					
> = 50	55 (41)	**2.41**	1.19–4.87	0.014	3.39	1.39–8.27	0.007
**Education level (*n*= 176)**							
High-school diploma	115 (62)	**1**					
No high-school diploma	61 (45)	**2.40**	1.22–4.74	0.011			
**Employment**							
Employed	93 (47)	**1**					
Unemployed	86 (62)	**2.53**	1.36–4.71	0.003	2.40	1.11–5.20	0.027
**Having children**							
No	89 (45)	**1**					
Yes	90 (64)	**2.41**	1.30–4.46	0.005			
**Living in own home (*n*= 177)**							
Yes	136 (82)	**1**					
No	41 (26)	**1.14**	0.55–2.35	0.720			
**Living alone**							
No	102 (57)	**1**					
Yes	77 (52)	**1.64**	0.89–3.04	0.115			
**Socializing with heavy drinkers**							
Yes	95 (48)	**1**					
No	84 (61)	**2.60**	1.39–4.86	0.003	3.84	1.66–8.85	0.002
**Tobacco Smoking**							
Non-smoker	58 (20)	**1**					
Regular smoker	121 (89)	**5.28**	2.69–10.38	0.000	9.72	3.91–24.15	0.000
**Binge drinking**							
Less than once a month	18 (8)	**1**					
Once a week	49 (11)	**0.36**	0.11–1.14	0.082			
Every day or almost every day	112 (90)	**5.11**	1.81–14.47	0.002			
**Had an alcohol-related health problem**							
No	80 (33)	**1**					
Yes, slight problem(s)	60 (41)	**3.07**	1.52–6.21	0.002			
Yes, serious problem(s)	39 (35)	**12.46**	4.04–38.43	0.000			
**Felt discriminated against because of alcohol consumption**							
No	94 (44)	**1**					
Yes	85 (65)	**3.69**	1.94–7.04	0.000	2.35	1.10–5.05	0.028
**Quality of patient-general practitioner relationship (*n*** **= 166)**							
Excellent or good	126 (74)	**1**					
Average or poor	40 (26)	**1.31**	0.62–2.74	0.481			
**Aware that AUD care no longer necessitates abstinence**							
No	102 (62)	**1**					
Yes	77 (47)	**1.01**	0.55–1.85	0.972			
**Use of internet-based medical forum**							
No	149 (87)	**1**					
Yes	27 (19)	**1.69**	0.70–4.11	0.245			

However, variables related to patients’ relationships with their general practitioner and their awareness that AUD care no longer necessitates abstinence, were not significantly associated with the outcome (Table 4).

#### Multivariable analysis

After multiple adjustment (Table 4), the following five variables remained associated with an increased likelihood of seeking/receiving care (*p* < 0.05): being over 50 years old (adjusted odds ratio [95% confidence interval]: 3.39 [1.39–8.27]), unemployment (2.40 [1.11–5.20]), not socializing with heavy drinkers (3.84 [1.66–8.85]), regular tobacco smoking (9.72 [3.91–24.15]), and having already experienced discrimination because of alcohol consumption (2.35 [1.10–5.05]). All VIF values were ≤ 1.5.

### Qualitative substudy results

#### General characteristics of study population

Among the 36 participants in the qualitative substudy, 15 (41.6%) were female. One (2.7%) participant was born between 1940 and 1949 (age 79–88), 4 (11.1%) between 1950 and 1959 (age 69–78), 16 (44.4%) between 1960 and 1969 (age 59–68), 7 (19.4%) between 1970 and 1979 (age 49–58), 6 (16.6%) between 1980 and 1989 (age 39–48), and 1 (2.7%) between 1990 and 1999 (age 29–38). Thirty (83.3%) participants were receiving AUD care at enrolment while 6 (16.6%) had not sought/received care for this specific disorder.

#### Results from the descending hierarchical classification

Five lexical field classes were defined using Iramuteq’s descending hierarchical classification, and were categorized into themes separately by two researchers to ensure the reproducibility of results. They then discussed their respective findings together to choose the titles for these emerging themes. Table 5 presents an overview of the results. Class 1 included 932 text segments (24.68%) which we named “drinking context”, Class 2 (295 text segments (25.29%)) “medical care”, Class 3 (429 text segments (11.36%)) “alcohol treatment”, Class 4 (661 text segments (17.51%)) “tobacco/addiction”, and finally, Class 5 (799 text segments (21.16%)) “family”.

**Table 5:** Descending Hierarchical Classification Results, qualitative study (*n* = 36)

Class characteristics		Associated Variables (*p* < 0.05)	Theme	Examples of associated words
Class 1	932 text segments (24.68%)	-Year of birth 1950–1959 - AUDIT score: < 16 and 19> - Experiencing health issues - Having one dependent child	Drinking context	To drink – Drink – Beer – Evening – Wine – Morning – Bottle – In the evening – Day time – Pub – Friends – Whisky – Noon – Table - Aperitif
Class 2	295 text segments (25.29%)	- Female gender - Receiving medical care - Having no dependent child	Medical care	Appointment – Hospital – Physician – Rehab – Clinic – Nurse – Psychologist – Month – Centre – To talk – Group – Workshop
Class 3	429 text segments (11.36%)	- Female gender - Receiving care - Severe AUD (AUDIT score ⩽ 20) - Year of birth 1960–1969 and 1970–1979 - Tobacco consumption - Being employed	Alcohol treatment	Drug – Abstinence – Baclofen – Antidepressants – Selincro – Treatment – Aotal – To consider – “Doctor’s name” - Total - Control
Class 4	661 text segments (17.51%)	- Receiving care - Having no children - Year of birth 1970–1979 - Tobacco consumption - Being employed	Tobacco and other addictions	To smoke – To stop – Alcohol – Cigarette – Consumption – Effects – Cannabis – To perceive – Drug (illicit) – To crave – Cocaine – To reduce – Joint (Cannabis cigarette)
Class 5	799 text segments (21.16%)	- Male gender - Being single - Year of birth 1950–1959 - Being unemployed - Having more than one dependent child	Family	Family – Child – To lose – Mother – Father – Daughter – Brother – Wife – Couple – Parents – Relationship – Death – To kill

#### Description of qualitative results

##### Triangulation of qualitative and quantitative findings

Triangulating data from the logistic regression and the textual analysis, two barriers (social drinking and difficulties with the medical care system), and two levers (family influence and tobacco addiction) emerged:

#### Barriers to seeking/receiving care for AUD

##### Socioenvironmental barriers

In the multivariable analysis, PWAUD who did not socialized with heavy drinkers and those unemployed were, respectively, almost 4 times (3.84 [1.66–8.85]) and 2 times (2.40 [1.11–5.20]) more likely to have received or sought care to stop or reduce their alcohol consumption.

The verbatim transcripts seemed to highlight that being surrounded by people who drink was itself an incentive to drink. Excerpts below illustrate this trend:

*"My husband always drinks a beer or two at noon, as an aperitif; in the evening, he always drinks one, two, or more glasses of vodka or rum …."*

*"I went to hang out with my colleagues, I gambled a little, and there I was, with everyone drinking, and I said to myself I was going to have a shot, it doesn’t matter if I have one, two..."*

*"Actually I started drinking around twenty, with friends, in the evening, and it started with beer, without getting drunk, but we hung out in the garden and we started to drink."*

#### Difficulties related to the medical care system

In the statistical analysis, the quality of the relationship with one’s general practitioner was not associated with AUD care. Furthermore, the qualitative study revealed that the process of accessing medical care was never linear: patients had to deal with several health professionals from different services and structures which referred them or re-referred them to other services, and this sometimes constituted an impediment to entering care.

*“For now, I get help from the addiction centre; then, if I notice that it gets worse, that instead of decreasing, my consumption increases, I’ll ask the doctor so I can go back to rehab.” (In care)*

*“I had made an appointment with a nurse at the time and I explained to her, but I was still drinking, so she said to me: ‘Well, listen, if you want, we’ll send you to rehab ...’, but I never went back to her.” (In care)*

In addition, some participants reported not feeling comfortable with certain dimensions of care, such as support groups.

*“It’s not that I don’t like the support group, it’s just that we always hear the same things, they’re always complaining over there.” (In care)*

Moreover, patients considered that using pharmacotherapy was a complex process: they had to try several drugs before finding the most appropriate therapy:

*“No, I take Selincro, as well as drugs to cure alcohol abuse and antidepressants. First, I was given Aotal three times a day, and then I started Selincro.” (In care)*

Additionally, PWAUD with comorbidities often had to combine several pharmacological treatments, which in some cases hindered them from taking AUD pharmacotherapies:

*“Concerning medications, there is the one I currently have; I take two anti-depressants in the morning. I don’t take anxiolytics because here, I’ve quit benzodiazepines.” (In care)*

*“I already take so much medicine for HIV, thyroid, cholesterol ... so I was afraid to take another drug ... Too many side effects: I read the leaflet, and there are many of them.” (Not in care)*

Finally, the fear of developing another addictive behaviour was frequently cited as an impediment to agreeing to take AUD pharmacotherapies.

*“I would agree to take pharmacological drugs, but warily, because I’ve never taken either anxiolytics or antidepressants ... And I think it would be stupid to quit one addiction for another.” (In care)*

#### Levers to receiving/seeking AUD care

##### Tobacco smoking

In the multivariable analysis, tobacco smoking remained significantly associated with an increased likelihood of seeking/receiving care to reduce or stop alcohol consumption. Regular smokers were almost ten times (9.72 [3.91–24.15]) more likely to have received or sought such care.

Our qualitative data shed light on the interaction between tobacco smoking and alcohol. First, some people who had already successfully stopped smoking felt they might be able to do the same with alcohol. Furthermore, they indicated that even if their attempt did not work, it would in any case give them more confidence to try to stop drinking alcohol:

*“There are improvements in certain periods I noticed something quite interesting. Last December I had lung problems, I was hospitalized, suddenly I quit smoking, I stayed at the hospital for five days, I didn’t drink a drop, I didn’t feel any craving ( …*) *That’s exactly it; when I was in the hospital, I quit smoking, I used a nicotine patch for four days. I didn’t miss tobacco a lot, even though I was a very heavy smoker. When I left the hospital, I realised that I hadn’t smoked for four days and I didn’t miss it. It would have been stupid to start again. It’s not the same for alcohol, I had spent four days without drinking too, but when I got to my place, I went to buy a good bottle of wine, for the pleasure of it (...).”* (I.e. In this quote, “hospital” refers to hospitalization for a medical issue unrelated to alcohol, and does not refer to hospitalization for rehabilitation) (In care)

Second, the presence of both alcohol and tobacco consumption made some people feel they were poly-addicted, and this drove them to tackle their addiction issues. For those who were apprehensive about stopping both substances at once, participants indicated they would prefer to treat AUD first as they perceived it to be the more harmful of the two addictions.

*“It’s really on alcohol that I want to focus, because it’s difficult for me; it is the strongest dependence and the most harmful (...). Perhaps, if I kept smoking a little, it could help me to stop alcohol, I would feel that I’m not stopping everything all at once, I would be satisfied.” (In care)*

The first excerpt (i.e., *“That’s exactly it”*, etc.) does not support our quantitative result (people who successfully quit smoking were categorized as “non-smokers”), but suggests that tobacco addiction consultation could be an entry point to address AUD: the satisfaction of successfully quitting tobacco could represent an incentive to stop or reduce alcohol consumption.

#### Family

The theme of ‘family’ (lexical field Class 5) was present in many interviews. Family, especially children, was often an important incentive to seek care for AUD.

*“How did I realize that it was a problem? Because of the dizziness, the physical consequences, when I started to tremble, feeling depressed, my family’s suffering... When you hurt your daughter, your mother, your brothers and sisters, when they all come to you in tears... My sister raised the problem first, because we are very close, she made me come here to the centre” (In care)*

*“It was my family, my child, who made me consult health professionals, because they noticed that during family dinners, I lost control.” (In care)*

*“The first time, no, it was the doctor who decided to send me to the psychiatric hospital, the other two times, yes, it was my wife who called the emergency service and who sent me to the psychiatric hospital …*” *(In care)*

In the multivariable analysis, people who had already experienced discrimination because of their alcohol consumption were twice as likely (2.35 [1.10–5.05]) to have already sought or received AUD care. Importantly, discrimination mainly came from family members (data not shown).

Although we did not use an implementation science model, we formulated different hypotheses which, if confirmed during our study, might provide useful indications for improving access to care for AUD. These hypotheses were based on the possible effect of various factors (such as experiencing discrimination, social contact with other alcohol consumers, and seeking/receiving care for tobacco or other substance use disorders) on access to and engagement in AUD care.

The qualitative study enabled us to provide greater detail about the specific barriers and levers not revealed by the quantitative study. An example of the former was the complexity of the care trajectory, as revealed by the numerous healthcare professionals required for AUD care. An example of the latter was the importance of tobacco cessation programs as an entry point for AUD care, as they are likely to be less stigmatising than programs offered for AUD.

## Discussion

To our knowledge, this is the first study to use a mixed-methods approach to investigate barriers and levers to receiving and seeking AUD care among PWAUD in France. In contrast with our hypothesis, we did not find a positive association between “awareness that AUD care no longer necessitates abstinence” and the outcome. The convergence of the results from the two substudies (quantitative and qualitative) provided us with important information on PWAUD decision-making regarding care.

In both substudies, a social drinking environment appeared to be a barrier to care. More specifically, our quantitative data showed that not socializing with heavy drinkers was associated with the outcome. In the qualitative substudy, “drinking context” was the second most discussed theme in the textual analysis. Furthermore, the qualitative data highlighted that living with a drinker made alcohol reduction or cessation more difficult.

The cultural norm of drinking alcohol in France, especially wine, appears clearly in the textual analysis, as does the social function of alcohol. Together, they may constitute a barrier to seeking/receiving care. More specifically, as drinking is considered normal in the country, people are less likely to question their own consumption.

Social environment is known to influence alcohol consumption [18, 37, 38]. For example, among married couples, alcohol consumption may increase if a partner drinks [39]. As previously suggested, our results highlight that a better understanding of the social factors influencing alcohol use is needed to improve prevention of AUD and access to related care. Indeed, alcohol consumption in France is a double-edged sword: on the one hand, it is socially encouraged, while on the other, a person who drinks too much is heavily stigmatized. Someone who knows his/her limits as regards consumption is considered an “enthusiast”, whereas someone who drinks too much is viewed negatively. The line between these two opposing perceptions is very thin indeed [40].

In the quantitative substudy, having already experienced discrimination because of AUD (which mainly came from family members) was a predictor of receiving/seeking care to stop or reduce one’s alcohol consumption. This is consistent with the fact that in the qualitative substudy, the theme ‘family’, (e.g., wishing to protect family members from the consequences of AUD) appeared to be a lever for the outcome. The desire to improve familial relationships is known to be an incentive to seek medical care for alcohol consumption [41], and greater collaboration with family members could be an option to improve related care in PWAUD. While not prominent in the qualitative substudy, we found that having experienced discrimination (or stigmatization) related to alcohol consumption was associated with receiving and/or seeking AUD care. Although a similar mechanism was identified among tobacco smokers in one study [42], the opposite effect was observed in another study focusing on people with AUD in the United States [43].

Our results highlighted that older age (> 50) was associated with an increased likelihood of seeking/receiving treatment. Consistent with some previous studies [18, 44], they nevertheless contradict findings from at least one other study [45].

There are many possible explanations for the association between older age and seeking AUD treatment. Older people are more likely to have a longer alcohol history, and therefore are more likely to seek treatment [46]. There are also metabolic reasons which make alcohol use more dangerous for older patients [47] and could constitute a motivation to change one’s level of alcohol consumption. Data concerning AUD and treatment among older adults in France are lacking, despite the fact that the French population is aging [48]. Consequently, there is an urgent need to explore this issue.

For some of the study population, the unexpected association between unemployment and seeking care for AUD might be attributable to job loss or difficulties finding a job due to alcohol consumption. This contrasts however with previous studies which found a positive association between job loss, unemployment and AUD [49, 50], especially among people with a higher education level [51].

The absence of any association between the general practitioner-patient relationship and access to care contrasts with another study which reported the importance of this relationship in helping to address alcohol issues during routine medical consultation [52]. However, our study’s result could suggest that the large number of different health professionals involved when accessing care for the first time, diluted the role of the general practitioner-PWAUD relationship.

With regard to the complexity of accessing care, it worth remembering that healthcare expenses for all individuals living legally in French territory are covered by the universal public health insurance system [53]. Moreover, general practitioners (GP) in France already manage substance use disorders. For example they can prescribe opioid maintenance treatment [54]. Consequently, it may be possible to simplify AUD treatment initiation and orientation through the GP network, by increasing their awareness of the importance of AUD screening and management.

Our quantitative results highlighted that being a regular smoker was associated with a greater likelihood of seeking/receiving care. This runs counter to previous studies’ findings which found a negative association between tobacco smoking and alcohol treatment participation [55, 56].

Furthermore, our qualitative data highlighted the double role of tobacco cessation initiatives and programs. First, they constitute an opportunity to start AUD (as confirmed by the quantitative analysis where smokers were more likely to have received or sought care). It is possible that smoking cessation as an entry point for AUD care may be less stigmatising than *programs offered for AUD* [57]. Second, successful smoking cessation increases motivation to seek care for AUD: people who successfully quit smoking in our study reported that this was an incentive to stop or reduce alcohol consumption. A previous article on physicians’ attitudes to care for AUD also found a correlation between promoting smoking cessation programs and care for AUD [58]. These results illustrate not only the value of mixed-methods research [59] but also that studies targeting both providers and patients can better highlight how prioritizing interventions for smoking cessation can result in multiple benefits for other substance use disorders.

Our qualitative findings revealed that PWAUD may be reluctant to add another medicine to their current daily medication schedule. This result is in line with previous studies showing that pharmacotherapy is not a preferred treatment for AUD [60].

The theme of family was prominent in textual analysis and the verbatim transcripts showed that some participants decided to enter into care because of family pressure. It has been demonstrated elsewhere that people who are assigned to care by their family are more likely to aim at being abstinent [61]. Consequently, we can suppose that people who seek care to stop, not to reduce, their alcohol consumption, do so to please their family. This could explain why we did not find a positive association between “awareness that AUD care no longer necessitates abstinence” and "having received/sought for AUD care.

Our study has limitations. First, as recruitment was not only carried out in healthcare facilities (a decision to ensure that those excluded from care would be reached), we were unable to recover data on possible clinical diagnosed comorbidities (e.g. liver disease) which could be predictor of access to care for AUD.

Moreover, some of the participants recruited in the two bars may have been under the influence of alcohol while they took the survey. However, we decided to include this specific population in the study, as it was the only possibility to reach PWAUD not receiving medical care for AUD.

In our outcome, we combined those who sought care, but had not received it (which was a very limited group) with those that had both sought and received it. These two groups could conceptually be characterised as being different. Nevertheless, we preferred to combine them as we considered that seeking care is a necessary condition to engage in a process of reduction/cessation of alcohol consumption.

Furthermore, with regard to logistical reasons, recruitment took place in healthcare structures and bars in order to obtain two groups which could be compared and contrasted (those that had already sought or received care vs. others). Consequently, external validity is not guaranteed.

Another limitation is that all our data, including data on alcohol consumption, were based on self-reports which may be subject to social desirability bias. Nevertheless, previous research has already proven the validity of self-reports in alcohol-using populations [62]. Finally, the backward elimination procedure used in the quantitative study constitutes a limitation as collinearity between the predictors is not taken into account. However, variables were selected according to a pre-existing hypothesis and collinearity was tested for. We can therefore be confident that major pitfalls were avoided.

## Conclusion

One of the most important findings of this study is the difficulty in accessing medical care for AUD which can sometimes constitute an impediment to reducing or stopping one’s alcohol consumption. This highlights the urgent need for simplified and novel comprehensive care trajectories to reduce the clinical and public health burdens of AUD.

Our results underline the need for interventions targeting families and the social drinking network to increase awareness about AUD and related care. Greater emphasis should be placed on non-pharmaceutical approaches to reduce alcohol consumption, and on the delivery of clearer and more complete information regarding reduced drinking as a real therapeutic goal.

## Data Availability

The datasets generated and analysed for the current study are available from the corresponding author on reasonable request.

## Abbreviations

ASIA: Access to Care and Indifference toward Alcohol
AUD: Alcohol Use Disorder
AUDIT: Alcohol Use Disorder Identification Test
CISIH: Information and care centre for HIV
CSAPA: Medical centre for drug and alcohol dependence
ELSA: Addiction care liaison team
GP: General Practitioners
HIV: Human Immunodeficiency Virus
PWAUD: People With Alcohol Use Disorder
